# Health Tract, No. 347

**Published:** 1868-11

**Authors:** 


HEALTH TRACT, NO. 347.

### WARM WATER,

For its medicinal and curative effects has more value than half the drugs on the shelves of the apothecary, and the mode of applying it properly so as to derive from it the highest advantage, deserves to be deeply impressed on the mind of every one who has a desire to be useful in emergencies, to say nothing of the advantages which may result personally to the operator or to some other member of the family.

If your hands are very cold, *paddle* them in moderately warm water patiently until the numbness disappears and there is a feeling of comfortableness in them; this not only warms up the hands pleasurably, but imparts an agreeable warmth to the whole body, without allowing chills to run over you, as will sometimes be the case in holding the hands to the fire, scorching the face, burning the eyes and occasionally setting fire to the clothing.

If the feet are cold to any degree short of being frozen, it is a most delightful sensation, not only to the feet themselves, but to the whole body, to put them half leg deep, in luke-warm water, then add a little hotter water every minute or two; at the end of ten or fifteen minutes, there will be experienced a degree of comfortableness throughout the whole body, which will amply repay the trouble taken.

If you are very warm, perspiring profusely, and are, very properly, afraid of cooling off too quickly, fill a basin three parts full of warm water, and paddle the hands in and out; the layer of water touching the skin is converted into steam the moment the hand is out of the water, thus causing a rapid carrying off of heat by evaporation; such an operation is perfectly delightful when the feet and hands are dry and hot, or are burning with fever.

There is scarcely an ache or pain in the whole body which is not soothed or removed by hot water, if applied as follows: dip a piece of flannel (or any cloth) of five or six folds or layers, in boiling water, and lay it on the painful part, covering it instantly with a dry flannel whose edges extend over the wet one an inch or more; as soon as the wet flannel has dried a little, say in five minutes or less, slip it out under one edge of the dry cloth, and introduce another flannel as hot as can be handled; do this in so adroit a manner as to allow as little cold air as possible to get to the skin touched by the hot flannel; keep on thus, until the pain or other suffering is removed; the most violent, dry and distressing coughs are thus loosened in a few minutes by this hot water poultice being applied to the throat and upper part of the chest; if done adroitly, the worst forms of croup in children are so subdued in half an hour, that the child, which was almost gasping for breath, perspires, its cough loosens, and it falls asleep. If the outer clothing has not been allowed to get damp as soon as sleep comes, wrap up the child warmly and keep it perspiring for several hours; but always send for a physician the instant a child is attacked with croup or sore throat, meanwhile do as above; and if a doctor cannot be had, see that the feet and hands are kept warm, and give a teaspoon of syrup of Ipecac or half as much hive syrup, every 15 or 20 minutes, to induce vomiting.

